# A Case of Organic Acidemia: Are Physicians Aware Enough?

**DOI:** 10.5005/jp-journals-10018-1175

**Published:** 2016-07-09

**Authors:** Taskina Mosleh, Sanjoy Kumer Dey, Md Abdul Mannan

**Affiliations:** 1 Department of Neonatology, Bangabandhu Sheikh Mujib Medical University, Dhaka, Bangladesh

**Keywords:** Fatal prognosis, Inborn error of metabolism, Organic academia.

## Abstract

Diversity of clinical presentation of inborn errors of metabolism (IEM) gives a diagnostic challenge to the practicing physicians. In recent years there have been dramatic advances in the diagnosis and treatment of these fatal diseases, which shows improved prognosis of many of these conditions. The need for screening for IEM arises out of the fact that most cases take to irreversible effects as time progresses. Here, the main challenge is to recognize the early signs and symptoms that are also common to sick infants with other diseases. We describe a 32-day-old female infant who was finally diagnosed as a case of IEM (organic acidemia).The baby had a history of multiple neonatal intensive care unit admission. She died during her last hospital admission. The purpose of our case presentation is to provide clues to the true nature of the disease and to make physicians aware about the possibility of IEM.

**How to cite this article:**

Mosleh T, Dey SK, Mannan MA. A Case of Organic Acidemia: Are Physicians Aware Enough? Euroasian J Hepato-Gastroenterol 2016;6(1):89-90.

## INRODUCTION

Inborn error of metabolism (IEM) is an inherited enzyme deficiency leading to the disruption of normal body metabolism that causes accumulation of a toxic substrate (a compound acted upon by an enzyme in a chemical reaction) or impaired formation of a product normally produced by the deficient enzyme.^1-3^Although it is individually rare, inborn error of metabolisms all together constitute a significant health problem causing increasing mortality and morbidity in infants.^4^ Diseases caused by IEM are constantly growing due to new identification techniques, but their incidence is not increasing in parallel. Probably, since it is considered rare, many physicians do not consider IEM until most frequent conditions have been ruled out. Here we report a rare case of organic acidemia with its clinical course.

## CASE REPORT

A 32-day-old female infant, 2nd issue of its nonconsan-guineous parents, was referred from a private hospital to the neonatal intensive care unit (NICU) of Bangabandhu Sheikh Mujib Medical University (BSMMU), Dhaka, Bangladesh, due to respiratory distress and less activity for 1 day. She also had feeding intolerance characterized by emesis 5 to 10 times a day, mostly following feeding since 11 days of age.

The baby was delivered by a lower uterine cesarean section (LUCS). Her mother is 34 years old, G5 P2 Ab2 magnetic resonance imaging (MR1), blood group O+ ve, on regular antenatal check-up, had a history of diabetes mellitus, but no fever, rash, or any other chronic illness. In the last trimester she developed hypertension, for which LUCS was done at 36 weeks of pregnancy in a private hospital.

She gave birth to a female child weighing 1750 gm who cried immediately after birth with APGAR score of 7 at 1 minute and 9 at 5 minutes. As the baby was preterm with low birth weight, she was transferred and admitted up to 9th postnatal day in the NICU of the same hospital. After discharge, she had no complaint for 2 days at home and was nursed without difficulty. At 11th postnatal age she developed vomiting containing milk, after almost every feed. The baby was again admitted to a private hospital. During the hospital stay of 14 days, she developed hypoglycemia, convulsion, and features of sepsis and was treated with IVF, IV antibiotics, IV phenobarbitone, IV fosphenytoin, and IV NaHCO_3_.

On arrival at the NICU of BSMMU on day 32, the baby was lethargic and emaciated. The following were noted: SPO_2_: 90% at room temperature; vitals: heart rate 134/minute, RR 58/minute, temperature 36.0°C, CRT < 3 seconds, CBG 3.2 mmol/L; anthropometry: OFC 30 cm, wt 1550 gm, length 35 cm. Her head, ears, eyes, nose, and oropharyngeal structures were without obvious abnormalities. Her lungs were clear, but notable intermittent grunting was present. Her heart was normal with no murmurs. Her abdomen was flat and soft, but liver palpitation of 2 cm was observed. Extremities were normal, with 1+ pulses. Genitalia had a normal female pattern. Her reflex activity was poor. Other findings were unremarkable.

Empiric antibiotics were started considering sepsis in mind after sending sepsis workup. Her ABG showed severe metabolic acidosis with an increased anion gap of 18. Bicarbonate infusion was initiated to treat the acidosis.

The CBC report showed the following: Hb 13.5 gm/dL, total WBC 5730/mm^3^, DC-neutrophil 15%, lympho 74%, platelet count 1 lac, CRP positive, blood C/S no growth.

During her hospital stay we failed to continue feeding the baby due to repeated vomiting. On day 36 the baby again developed generalized seizure. Surgical causes were excluded. The baby most likely had a defect in metabolism. A metabolic defect workup was done. Serum ammonia and lactate level was elevated. Urine for ketone body was suggested, but parents refused to do the test. IMD (inborn metabolic disease) panel revealed acyl carnitines. Echocardiogram report showed dilated cardiomyopathy.

Treatment was started with Biotin and Levocarnitine. She later developed bradycardia, sepsis with DIC-like features. Her condition was worsening despite all efforts, and required mechanical ventilation. As her condition was not improving, a decision was made by her parents not to do any further invasive procedure as her condition was believed to be terminal; she died at the age of 55 days.

## DISCUSSION

Inborn error of metabolism causes a group of hereditary metabolic diseases resulting from a lack of activity of 1 or more specific enzymes or defects in the transportation of proteins resulting in a block of metabolic pathway.^1,2^ The frequency of occurrence of organic aciduria is 1 in 15,000 according to Seymour CA et al.^5^ The term “organic acidemia” or “organic aciduria” (OA) applies to a group of disorders characterized by the excretion of non-amino organic acids in the urine.^6^

Most organic acidemias become clinically apparent during the newborn period or early infancy.^7^ After an initial period of well-being, affected children develop a life-threatening episode of metabolic acidosis characterized by an increased anion gap. This presenting episode may be mistaken for sepsis and, if unrecognized, is associated with significant mortality.^1,2,6,8^ In our case as well we faced the same difficulties. The usual clinical presentation includes vomiting, poor feeding, neurologic symptoms such as seizures and abnormal tone, and lethargy progressing to coma.^2,9^ Appropriate laboratory testing for metabolic disorders should be performed in any infant who exhibits these findings.^8^

The exact diagnosis depends on specialized enzyme assays and/or the identification of molecular defect. These methods are not very widely available, especially in our country. Such tests are, however, suitable only when there is a strong and more specific suspicion of IEM diagnosis. In our context we mainly depend on the high index of suspicion.

## CONCLUSION

An IEM could be a diagnosis of inclusion rather than exclusion. It is frequently underestimated by the doctors in neonatal and intensive care units of national health clinics or in private clinic. An increase in the rate of identification of these disorders is directly related to clinical judgment and a lifesaving difference can be made by appropriate and timely care for the small number of babies with 1 of these disorders.
